# Discussion of Perspectives on Various Innovative Statistical Methodologies for Alzheimer’s Disease Clinical Trials

**DOI:** 10.1002/alz70859_110767

**Published:** 2025-12-26

**Authors:** John Lawrence

**Affiliations:** ^1^ Division of Biometrics I, US Food and Drug Administration, Silver Spring, MD USA

## Abstract

**Background:**

Innovative statistical methodologies have been proposed to improve Alzheimer’s clinical trial efficiency: (i) Models leveraging data across multiple post‐baseline visits might provide greater power compared to traditional MMRM approaches which focus on mean changes at the last visit. (ii) Machine learning techniques, such as those incorporating prognostic covariates or regression trees with fused leaves, may improve precision in treatment effect estimation. (iii) Alternative analyses such as time‐to‐event or ranking‐based approaches are becoming relevant as AD clinical trials increasingly focus on prevention and asymptomatic participants. This session seeks to stimulate a collaborative dialogue among regulatory agencies, Alzheimer’s associations, industry, and academia to evaluate the feasibility, benefits, and limitations of these methodologies in the context of AD clinical trials.

**Methods:**

This presentation will review the methodologies introduced by five speakers, formulating discussion questions around their statistical validity, challenges, and limitations in application and interpretability.

**Results:**

The session discussions are expected to deepen understanding of how innovative statistical approaches address the unique challenges of AD clinical trials. Insights gained may inform pathways for integrating novel methods into trial designs and analyses.

**Conclusions:**

Advances in our understanding of AD disease progression and treatment effects provide a opportunity to explore some innovative statistical methodologies. Rigorous evaluation and open dialogue are essential to utilize these methods effectively, ensuring they enhance trial efficiency and maintain the rigor required for regulatory decision‐making.

